# Advanced Optimization of Optical Carbon Dioxide Sensor Through Sensitivity Enhancement in Anodic Aluminum Oxide Substrate

**DOI:** 10.3390/polym17111460

**Published:** 2025-05-24

**Authors:** Manna Septriani Simanjuntak, Rispandi, Cheng-Shane Chu

**Affiliations:** 1 International Ph.D. Program in Innovative Technology of Biomedical Engineering and Medical Devices, Ming Chi University of Technology, New Taipei City 243303, Taiwan; mannasimanjuntak12@gmail.com; 2 Department of Mechanical Engineering, Faculty of Engineering, Universitas Andalas, Padang 25163, Indonesia; rispandi@eng.unand.ac.id; 3 Department of Mechanical Engineering, Ming Chi University of Technology, New Taipei City 243303, Taiwan; 4 Research Center for Intelligent Medical Device, Ming Chi University of Technology, New Taipei City 243303, Taiwan

**Keywords:** optical carbon dioxide sensor, AAO, CdSe/ZnS quantum dots, polymer, fluorescence

## Abstract

The current research developed an optical carbon dioxide (CO_2_) sensor using anodized aluminum oxide (AAO) as the substrate. We developed an optical carbon dioxide (CO_2_) sensor utilizing CdSe/ZnS quantum dots (QDs) as the fluorescent dye and Phenol Red as the pH indicator. The QDs acted as the CO_2_-responsive fluorophore and were embedded in a polyimide butyl methacrylate (polyIBM) matrix. This sensing solution was applied to an anodized aluminum oxide (AAO) substrate, which provided a porous and stable platform for sensor fabrication. Photoluminescence measurements were conducted using the coated AAO substrate, with excitation from a 405 nm LED light source. The sensor exhibited red fluorescence emission at 570 nm and could detect CO_2_ concentrations in the linear range of 0–100%. Experimental results showed that fluorescence intensity increased with CO_2_ concentration, achieving a sensitivity of 211. A wavelength shift of 0.1657 nm/% was observed, indicating strong interactions among CO_2_ molecules, Phenol Red, and the QDs within the AAO matrix. The sensor demonstrated a response time of 55 s and a recovery time of 120 s. These results confirm the effectiveness of this optical sensing approach in minimizing fluctuations from the excitation light source and highlight the potential of the AAO-supported QDs and Phenol Red composite as a reliable CO_2_ sensing material. This advancement holds promise for applications in both medical and industrial fields.

## 1. Introduction

The detection of carbon dioxide is critically important across diverse fields, including environmental monitoring, industrial oversight, and medical diagnostics. As a major contributor to global warming, CO_2_ significantly impacts climate change, highlighting the necessity for accurate and continuous monitoring to evaluate air quality and regulate emissions. In healthcare, precise CO_2_ sensing is vital for respiratory monitoring, ensuring patient safety during surgeries, and managing critical care scenarios [1,2,3,4]. Recent advancements in optical gas sensors have spurred substantial interest due to their wide applicability across multiple domains. These sensors offer transformative capabilities in gas detection, characterized by enhanced sensitivity, exceptional selectivity, and rapid response times. As the demand for reliable gas sensing technologies grows in areas such as environmental protection, healthcare, industrial operations, and clinical diagnostics, a variety of sensor technologies have emerged to detect specific gases, including carbon dioxide [5]. The primary methods for CO_2_ detection include infrared (IR) absorptiometry, solid-state electrochemical sensors, and optical sensing technologies [6]. Among these, optical CO_2_ sensors have gained widespread attention for their distinct advantages, such as electrical isolation, high fluorescence quantum efficiency, robust light absorption, superior photostability, and significant potential for miniaturization [7]. These sensors typically operate by detecting changes in fluorescence intensity, facilitated by pH-responsive dyes.

Recent advancements in optical CO_2_ sensors have introduced systems that utilize pH indicators exhibiting colorimetric or fluorometric changes, as well as CO_2_-sensitive dyes, providing improved sensitivity and selectivity [8]. Most pH-sensitive optical sensors function by detecting shifts in the fluorescence intensity of the pH-sensitive dyes, enabling highly sensitive and precise pH measurements [9]. Recent research has explored the use of colorimetric changes in pH indicator dyes such as thymol blue, α-naphtholphthalein [10], and Phenol Red, in addition to fluorescence variations in luminescent dyes, as core mechanisms for CO_2_ detection in gas sensor systems [11]. Moreover, polymer matrices such as PLA, PVA, and PolyIBM have been employed to create an optimal microenvironment for fluorescent molecules. These matrices stabilize the acidic or basic forms of the dyes, enhance gas diffusivity, and improve surface interactions with target molecules. This design promotes faster response and recovery times to gases like CO_2_, as the intrinsic permeability of polymers facilitates accelerated gas diffusion and fosters effective interactions with the target molecules [12,13,14].

Table 1 shows how several researchers have developed sensors based on colorimetric or optical methods for detecting carbon dioxide utilizing fluorescence intensity. The table highlights various designs, including sensors employing Cresol Red, Phenol Red, and Thymol Blue with the reference dye Eu(tta)_3_ in polystyrene, structured with double layers. These sensors exhibit low to moderate sensitivity (7.1–15.6) and primarily generate intensity-based signals. In contrast, a sensor combining α-naphtholphthalein with CIS/ZnS QDs in polyIBM utilizes a single-layer design and achieves significantly higher sensitivity (99.6), offering dual signal outputs through wavelength shift and intensity variations. Additionally, the pairing of α-naphtholphthalein with Tetraphenylporphyrin in a double-layer configuration demonstrates a wide sensitivity range (10.3–192), influenced by the supporting material. The sensor developed in this study, featuring Phenol Red and CdSe/ZnS QDs in polyIBM, achieves the highest performance among the compared designs. With a single-layer structure, it delivers an exceptional sensitivity of 211, measured through intensity-based signals. Notably, sensors employing CdSe/ZnS quantum dots with Phenol Red on an anodic aluminum oxide (AAO) substrate demonstrate superior responsiveness, achieving the highest recorded sensitivity (211). The anodic aluminum oxide (AAO) substrate offers distinct advantages over polymer matrices, especially in terms of thermal stability, mechanical strength, and nanoscale structural uniformity. Unlike polymers, which often degrade at elevated temperatures and can be chemically unstable, AAO is an inorganic material that remains stable under harsh thermal and chemical conditions [15]. Furthermore, AAO features a highly ordered and tunable nanoporous structure with uniform pore sizes, making it an ideal template for nanofabrication and surface patterning applications [16,17]. These properties provide a significant advantage for applications that demand precise control at the nanoscale, which is why AAO was selected in this study. The AAO substrate significantly enhances sensitivity and detection limits. Its perforated, nanoporous structure provides a high surface area, facilitating efficient transport of analyte molecules through the nanopores. This design maximizes interactions between CO_2_ molecules and the indicator molecules on the substrate’s surface, establishing AAO as an advanced material for optical carbon dioxide sensors [18,19].

In this study, we emphasized the sensitivity of optical CO_2_ detection based on colorimetric changes in Phenol Red, utilizing CdSe/ZnS quantum dots (QDs) as a fluorophore. CdSe/ZnS QDs were employed as the internal reference dye in a CO_2_-sensitive system, combined with Phenol Red as the pH indicator. The exceptional photophysical properties of CdSe/ZnS QDs, along with their significant spectral overlap between the emission spectrum of the QDs and the absorption spectrum of Phenol Red, make them highly effective as an internal reference dye. These innovations show great potential for enhancing CO_2_ detection accuracy in various applications, particularly those requiring non-invasive, real-time monitoring. Among the methods evaluated, drop coating CdSe/ZnS QDs and Phenol Red onto an anodic aluminum oxide (AAO) substrate proved to be the most sensitive. This combination effectively detects low concentrations of CO_2_, with high sensitivity favoring the detection of CO_2_ levels in the low ppm range. The AAO substrate, incorporated into the sensor design, significantly enhances sensitivity due to its nanoporous structure, which facilitates efficient CO_2_ diffusion and interaction with the indicator molecules. The colorimetric AAO-based sensor for optical CO_2_ detection developed in this research enables accurate detection across a wide range of concentrations, making it highly applicable to medical diagnostics, environmental monitoring, and industrial processes.

## 2. Experimental

### 2.1. Materials

CdSe/ZnS quantum dots (QDs), serving as fluorescent dyes, were sourced from Taiwan Nanocrystals Inc. (Tainan City, Taiwan), renowned for their high-quality nanomaterials. Phenol Red and PolyIBM were obtained from Alfa Aesar and Scientific Polymer Inc. (Heysham, Lancashire, Inggris, and United States) respectively, both well-known suppliers of research-grade chemicals. Tetraoctylammonium hydroxide (TOAOH), a critical reagent for nanoparticle synthesis, was procured from Sigma-Aldrich (Darmstadt, Germany) and further purified in-house using established protocols from the scientific literature to ensure high purity and reproducibility. Additionally, toluene, a commonly used solvent in nanoparticle synthesis, was purchased from Tedia Company Inc. (Fairfield, OH, USA) a reputable provider of high-purity laboratory chemicals.

**Figure 1:**
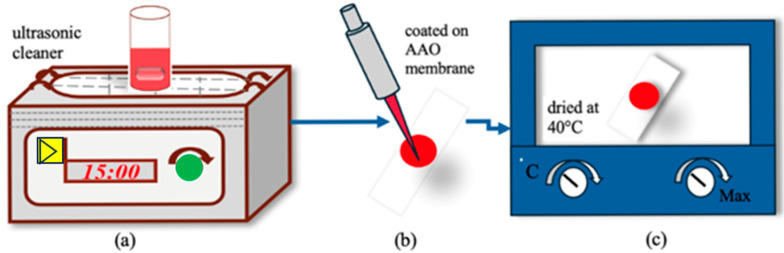
Process of preparing optical CO_2_ sensing materials: (**a**) solvent sonication using an ultrasonic cleaner, (**b**) coating of CdSe/ZnS QDs onto the AAO substrate, and (**c**) assembly of the optical CO_2_ sensor, followed by drying at 40 °C for 15 min with a 10-min holding period.

To prepare the optical CO_2_ sensor, an AAO substrate with a thickness of 60 μm is employed. The membrane is carefully placed on a glass substrate, which has been sterilized first with soap water for 15 min, followed by isopropanol for another 15 min. The glass is then air-dried at room temperature for 30 min. The glass substrate measures 4.4 cm in length and 1.3 cm in width. Once sterilized, the AAO substrate is positioned on the glass, and a fluorescent dye solution is uniformly coated onto the membrane in a volume of 30 μL. The assembled optical CO_2_ sensor is subsequently dried at 40 °C for 15 min, followed by a 10-min holding time. Upon completion of the drying process, the AAO substrate-based optical CO_2_ sensor undergoes gas sensing evaluation. Figure 1 illustrates the preparation process of the optical CO_2_ sensing materials.

### 2.2. Instrumentation

**Figure 2:**
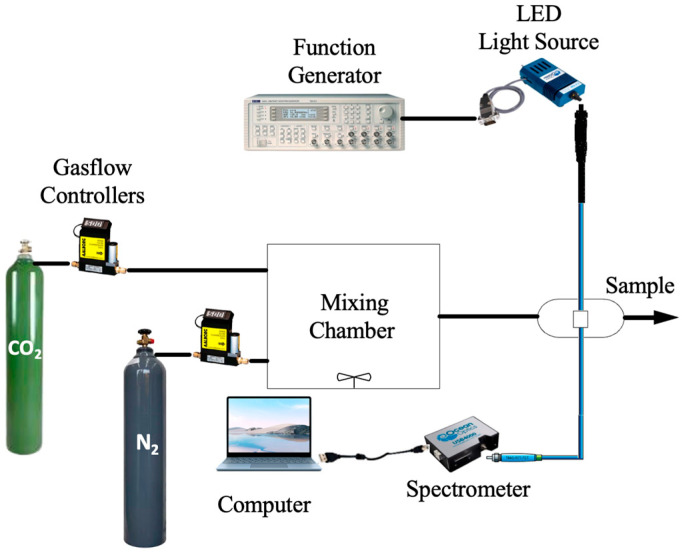
Schematic illustration of the CO_2_ gas sensing for analytical characterization.

Figure 2 illustrates the experimental gas setup used to collect data for carbon dioxide sensing trials. The LED lamp emits light at a wavelength of 405 nm, powered by a TGA1240 function generator (Thurlby Thandar Instruments Ltd., Huntington, UK), which operates with high precision and stability to ensure consistent illumination at a frequency of 10 kHz, enabling CO_2_ detection on the anodized aluminum oxide (AAO) substrate. The function generator ensures accurate modulation of the light source to optimize sensor performance. Fluorescence intensity, a key indicator of CO_2_ presence, was carefully measured using a USB spectrometer 4000, recognized for its high sensitivity and accuracy in spectral measurements. This setup facilitated reliable detection and analysis of CO_2_ based on fluorescence responses [20]. A mass flow controller was employed to adjust the concentration by blending pure CO_2_ and N_2_ gases in the mixing chamber before introducing the mixture to the sample holder. Data acquisition was conducted using Spectra Suite software (version 2024), followed by graphical analysis in MS Excel (version 2024). Surface morphology and elemental composition were meticulously analyzed using Field Emission Scanning Electron Microscopy (FESEM, JEOL JSM-6701) in conjunction with Energy Dispersive X-ray (EDX) Spectroscopy, enabling high-resolution elemental mapping and identification. Additionally, Transmission Electron Microscopy (TEM) was utilized to investigate the structural properties and size distribution of CdSe/ZnS quantum dots (QDs) on the AAO substrate, providing high-resolution imaging for precise characterization of nanoparticle dispersion and interactions at the nanoscale.

## 3. Results and Discussion

### 3.1. Morphological Characterization of CdSe/ZnS Quantum Dots via Scanning Electron Microscopy (SEM)

**Figure 3:**
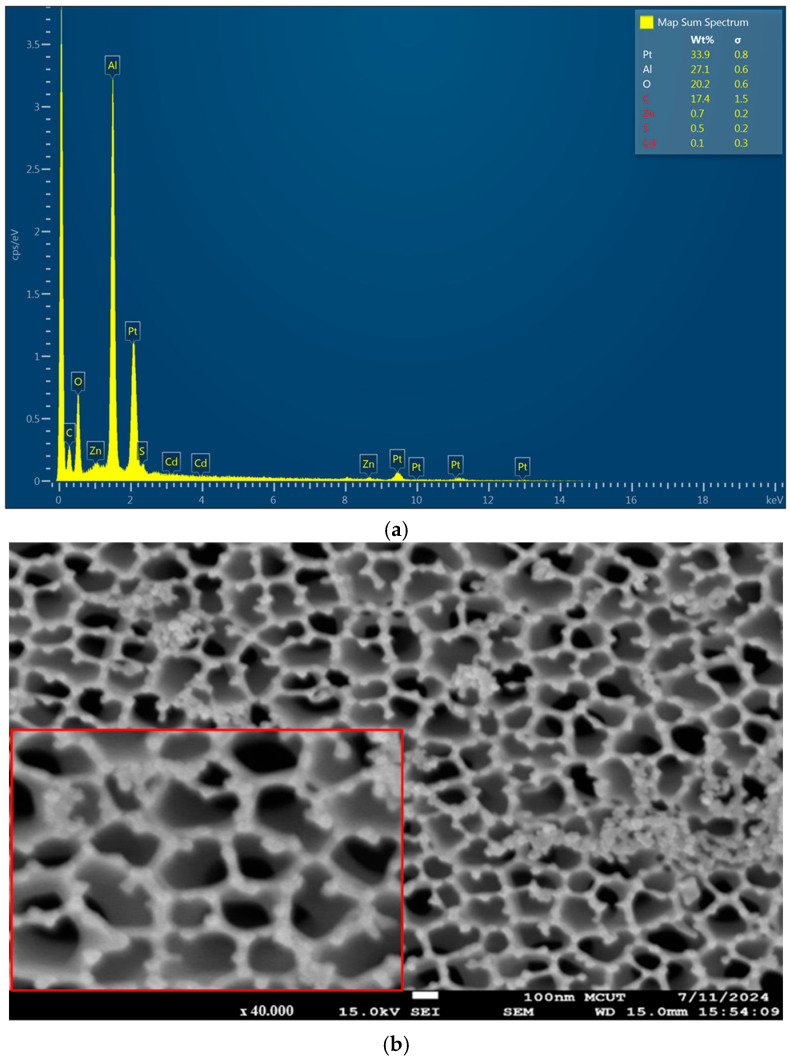
(**a**) SEM image showing the surface morphology of the AAO substrate embedded with CdSe/ZnS QDs at 10,000× magnification and (**b**) EDX analysis of the AAO substrate containing CdSe/ZnS QDs.

Figure 3 presents high-resolution Scanning Electron Microscope (SEM) images of an anodic aluminum oxide (AAO) substrate embedded with CdSe/ZnS quantum dots (QDs). Figure 3a shows a high-magnification SEM image (10,000×), highlighting the morphological features of the AAO substrate. The image reveals a characteristic honeycomb-like structure with pores approximately 1 µm in diameter, a distinctive feature of AAO substrates formed via electrochemical anodization. Figure 3b displays the Energy Dispersive X-ray (EDX) spectrum, which provides critical information on the elemental composition of the AAO substrate embedded with CdSe/ZnS QDs. The prominent peaks corresponding to Cd, Se, Zn, and S confirm the successful incorporation of the QDs into the membrane, with the relative intensities of the peaks indicating a substantial concentration of these quantum dots.

### 3.2. Fabrication of CO_2_ Sensing Material on AAO Substrate Characterized by CdSe/ZnS Quantum Dots via Transmission Electron Microscopy (TEM)

**Figure 4:**
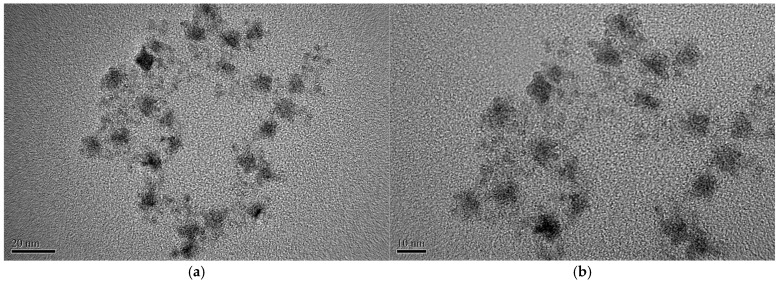
TEM images of CdSe/ZnS QDs, at resolutions of (**a**) 20 nm and (**b**)10 nm.

The optical CO_2_ sensor is fabricated using a supporting matrix material produced through a mixing process. To create the matrix, 200 mg of polyIBM powder is dispersed in 2 mL of toluene and agitated for 15 min with a magnetic stirrer until fully homogenized. For the fluorescent dye solution, 30 mg of CdSe/ZnS quantum dots and 3 mg of Phenol Red are dissolved in 300 µL of TOAOH as the solvent and sonicated for 15 min in an ultrasonic cleaner. The dissolved CdSe/ZnS quantum dots and Phenol Red are then combined with the previously prepared matrix. Finally, all components are stirred together with a magnetic stirrer for 1 h at 27 °C until the solution is thoroughly mixed. Figure 4 shows detailed transmission electron microscopy (TEM) images of CdSe/ZnS quantum dots, acquired at specified resolutions of 20 nm and 10 nm, indicating the nanoscale quantum dot diameter of roughly 6 nm.

### 3.3. The Absorption and Emission Characteristics of CdSe/ZnS QDs

**Figure 5:**
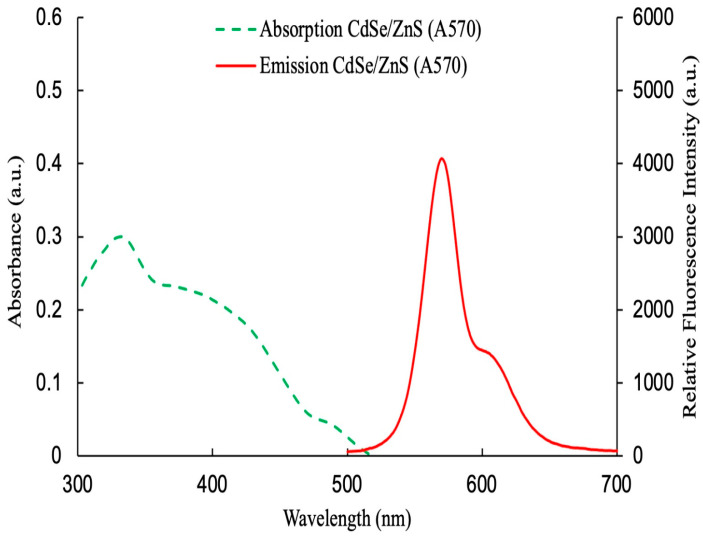
Absorption (dashed line) and emission (solid line) spectra of CdSe/ZnS QDs.

Figure 5 presents the optical properties of CdSe/ZnS quantum dots (QDs), characterized using a UV-Vis spectrophotometer and a USB4000 spectrometer, with excitation provided by a 405 nm LED source. The absorption spectrum (green dashed line) displays a broad profile with a notable decline in intensity beyond 500 nm, corresponding to the material’s bandgap edge. Upon excitation, the QDs exhibit a sharp and intense emission peak around 590 nm (red solid line), demonstrating a pronounced Stokes shift due to energy relaxation prior to photon emission. The high emission intensity indicates a superior quantum yield, reflecting efficient energy conversion from absorbed to emitted light with minimal non-radiative losses. The core–shell structure of CdSe/ZnS plays a critical role in enhancing photostability and suppressing surface defects, thereby supporting strong and stable fluorescence. These exceptional optical characteristics position the QDs as highly promising candidates for advanced applications in optoelectronic devices, high-resolution bioimaging, and next-generation photonic systems.

### 3.4. Cross-Section and Elemental Analysis of Anodic Aluminum Oxide Membrane

**Figure 6:**
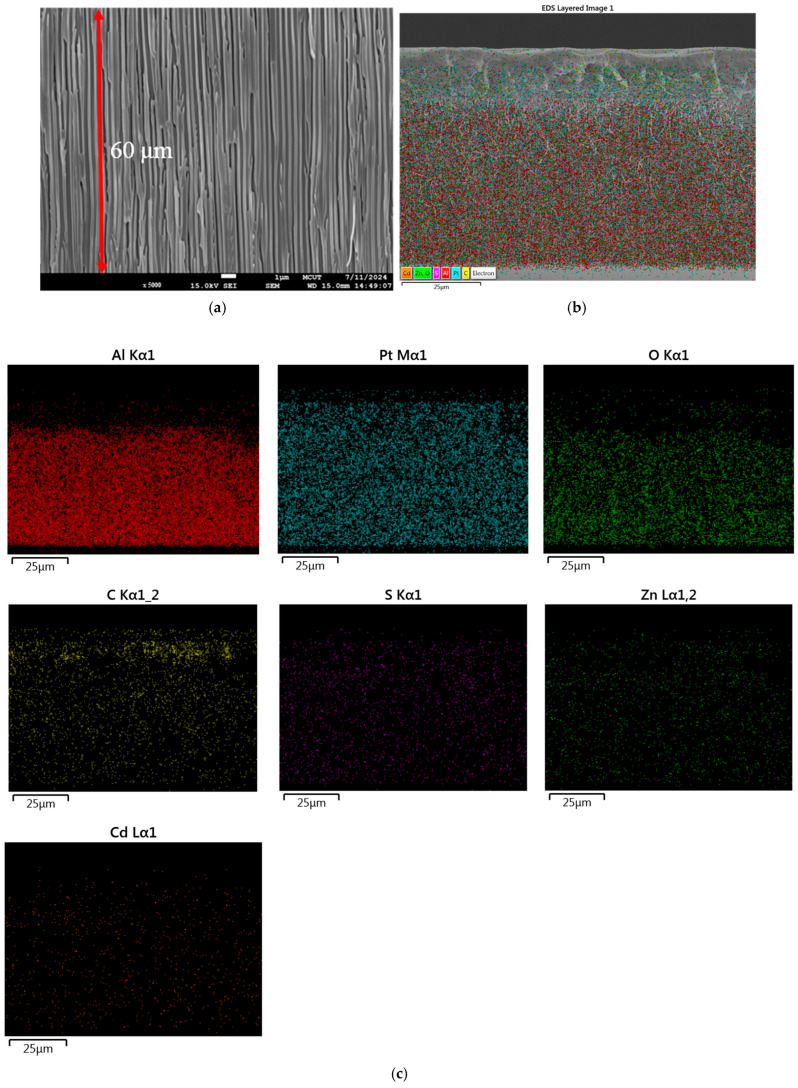
SEM characterization of the AAO substrate, including: (**a**) cross-sectional view of the AAO structure, (**b**) electron distribution analysis for elemental detection, and (**c**) EDX mapping of the AAO substrate for compositional analysis.

The morphological properties of the anodic aluminum oxide (AAO) substrate surface were analyzed using SEM. Figure 6a presents a low-magnification SEM image (5000×), providing a broad overview, including a cross-sectional visualization of the membrane. The red line indicates the membrane thickness, measured at 60 µm, which aligns with the typical range for AAO substrates fabricated via anodization. Figure 6b highlights an SEM image focusing on the spatial distribution of CdSe/ZnS QDs embedded within the AAO substrate. Furthermore, Figure 6c showcases the EDX mapping analysis, offering detailed elemental detection across the AAO substrate surface.

### 3.5. X-Ray Diffraction (XRD) Analysis of CdSe/ZnS Quantum Dots and AAO Substrate

**Figure 7:**
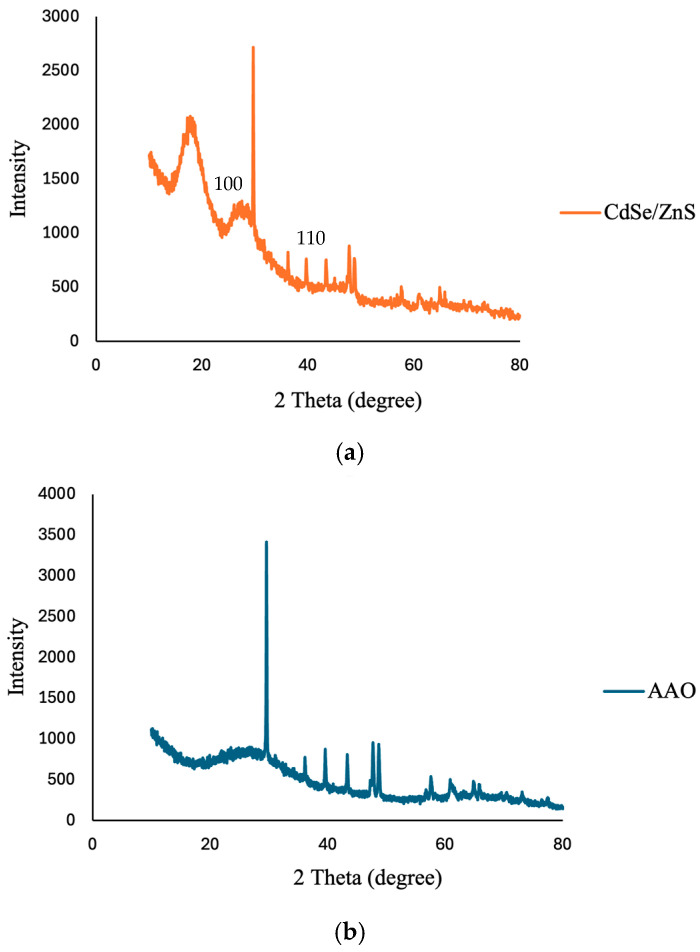
XRD characterization of the AAO substrate, (**a**) CdSe/ZnS quantum dots (QDs), (**b**) AAO membrane.

Figure 7 shows the X-ray diffraction (XRD) patterns of two samples: (a) CdSe/ZnS (orange curve) and (b) AAO (blue curve). The CdSe/ZnS sample exhibits several sharp and narrow diffraction peaks at distinct 2θ positions, notably around 25°, 30°, and 45°, which are characteristic of a well-defined crystalline structure. These peaks indicate that the atoms within the CdSe/ZnS sample are arranged in a highly ordered crystal lattice. In contrast, the AAO (Anodic Aluminum Oxide) sample displays a broad, diffuse hump in the 2θ range of approximately 45–60°, with no distinct sharp peaks. This suggests the material is amorphous, as it lacks long-range atomic order. The overall intensity difference also highlights the strong crystallinity of CdSe/ZnS compared to the more disordered structure of AAO.

### 3.6. Optical Carbon Dioxide Sensor Sensing Properties

**Figure 8:**
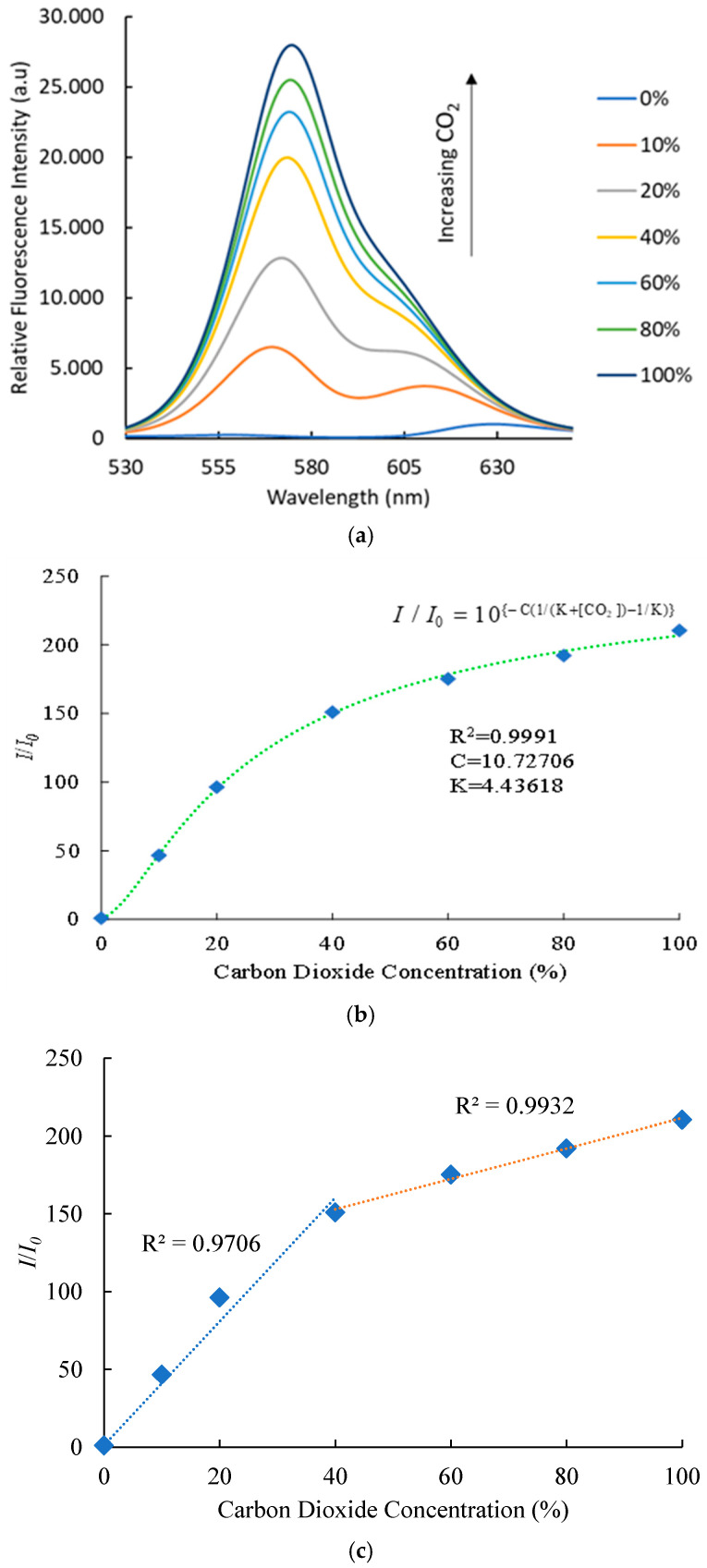
(**a**) Optical spectrum showing relative fluorescence intensity of the CO_2_ sensor at different CO_2_ concentrations, (**b**) calibration plots illustrating the CO_2_ gas sensing behavior, and (**c**) relationship between CO_2_ concentration and the fluorescence intensity ratio (*I*/*I*_0_).

Analysis of the fluorescence spectra of CdSe/ZnS quantum dots embedded within an AAO substrate reveals that the optical CO_2_ sensor demonstrates remarkable sensitivity and responsiveness to a wide range of CO_2_ concentrations. The luminescence spectra of CdSe/ZnS quantum dots were systematically examined over CO_2_ concentrations ranging from 0% to 100%. Upon excitation at 405 nm, the CdSe/ZnS QDs exhibited a well-defined fluorescence emission peak at 570 nm, characteristic of electronic transitions within the quantum dot structure. Figure 8a illustrates the relationship between increasing CO_2_ concentrations and the corresponding rise in relative fluorescence intensity, with a peak around 580 nm. As CO_2_ levels increase from 0% to 100%, the fluorescence intensity significantly enhances, indicating a strong correlation between CO_2_ concentration and luminescence output. This behavior is likely due to the interaction of CO_2_ with the CdSe/ZnS quantum dots, which modifies surface states and reduces non-radiative decay pathways, thereby enhancing radiative recombination. Additionally, the presence of Phenol Red, a pH-sensitive dye, may amplify the optical response through its colorimetric shift induced by CO_2_-driven pH changes. These findings underscore the optical sensor’s exceptional sensitivity to CO_2_ variations, demonstrating its potential for diverse applications.

The sensitivity to CO_2_-induced quenching is quantified by the ratio *I*_100*%CO*2_/*I*_100*%N*2_, where *I*_100*%CO*2_ and *I*_100*%N*2_ represent the fluorescence intensities in pure CO_2_ and N_2_ atmospheres, respectively [21]. In the absence and presence of CO_2_ gas, the fluorescence intensities corresponding to the luminescence sensing signals are denoted as *I_0_* and *I*, respectively. The calibration plot in Figure 8b illustrates the relationship between intensity ratios (*I*/*I*_0_) and varying CO_2_ concentrations, as described by Equation (1). The graph demonstrates the nonlinear relationship between carbon dioxide concentration ([CO_2_]) and intensity ratios (*I*/*I*_0_), modeled by Equation (1) with constants C = 10.72706 and K = 4.43618. The fitting results show exceptional agreement, with an R^2^ value of 0.9991, indicating that the model accurately reflects the experimental data. The optical sensor achieves a peak sensitivity of 211 at 100% CO_2_ concentration, underscoring its outstanding detection capability and precision. With this exceptional performance, the optical sensor is ideally suited for applications such as environmental monitoring and medical analysis, where high accuracy and sensitivity are critical.

(1) $$I/I_{0}=10^{\left\{-C(1/(K+[CO_{2}])-1/K)\right\}}$$

Figure 8c presents the calibration plot analysis of an optical sensor constructed using an AAO substrate embedded with CdSe/ZnS quantum dots. The plot reveals two distinct linear regions in fluorescence quenching behavior as a function of CO_2_ concentration. For concentrations between 0% and 40%, the fluorescence quenching exhibits a linear correlation with an R^2^ value of 0.9706. From 40% to 100% CO_2_ concentration, the quenching follows a separate linear trend with a higher R^2^ value of 0.9932, indicating robust sensitivity in both ranges. The optical CO_2_ sensor’s peak sensitivity, calculated to be 211, underscores its exceptional performance. Given its precision and sensitivity, this optical sensor holds significant potential for applications requiring accurate CO_2_ detection, such as indoor air quality monitoring and industrial process control.

### 3.7. Wavelength Shift of Optical Carbon Dioxide Sensor

**Figure 9:**
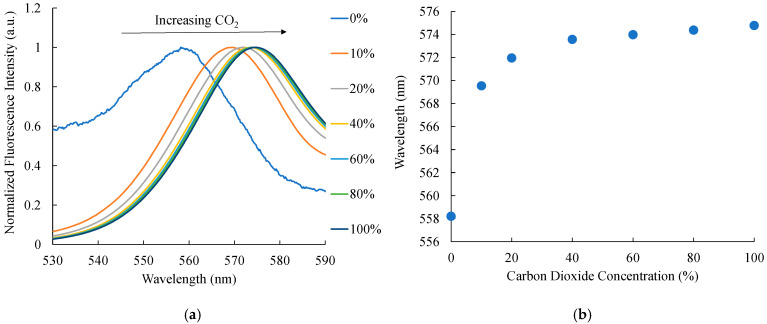
(**a**) Wavelength shifts of the carbon-dioxide-sensitive dye and (**b**) the correlation between wavelength shift and CO_2_ concentration.

Referring to Figure 9a, which illustrates the spectral wavelength shift with increasing carbon dioxide concentrations from 0% to 100%, a wavelength shift of 0.1657 nm per percent (nm/%) is observed. In this sensor, Anodic Aluminum Oxide (AAO) is employed as the optical sensor’s substrate, playing a vital role in CO_2_ detection. The addition of Phenol Red as a pH indicator also influences this wavelength shift. Dissolved CO_2_ forms carbonic acid, lowering the pH and causing chemical changes in Phenol Red. These changes modulate the optical environment around the CdSe/ZnS quantum dots (QDs) integrated into the AAO substrate, which affects the electronic properties and emission characteristics of the sensor, leading to a significant wavelength shift. The observed shift of 0.1657 nm/% indicates a strong interaction between CO_2_ molecules, Phenol Red, and QDs within the AAO substrate. The consistent shift pattern shown in the figure confirms the proportional relationship between CO_2_ concentration and wavelength shift. This validates that the method is effective in accurately and reliably detecting and measuring variations in CO_2_ concentration while overcoming the limitations of luminescence intensity measurements, which are susceptible to fluctuations in optical power.

Figure 9b shows the graph illustrating the correlation between carbon dioxide concentration and the optical response of CdSe/ZnS quantum dots. The peak emission wavelength demonstrates a redshift (towards longer wavelengths) as the CO_2_ concentration increases from 0% to 100%. This redshift signifies a reduction in photon energy, attributed to CO_2_-induced modifications in the quantum dots’ local environment, which decrease the energy gap between their excited and ground states. Simultaneously, the luminescence intensity of the quantum dots increases with the CO_2_ concentration, likely due to enhanced radiative recombination efficiency or the suppression of non-radiative pathways facilitated by the interaction between CO_2_ and the quantum dots. This dual response, involving shifts in wavelength and luminescence intensity, underscores the potential of CdSe/ZnS quantum dots as highly sensitive and robust optical sensors for CO_2_ detection, providing precise and reliable monitoring capabilities.

### 3.8. Response Time and Dynamic Characteristics of Optical Carbon Dioxide Sensor

**Figure 10:**
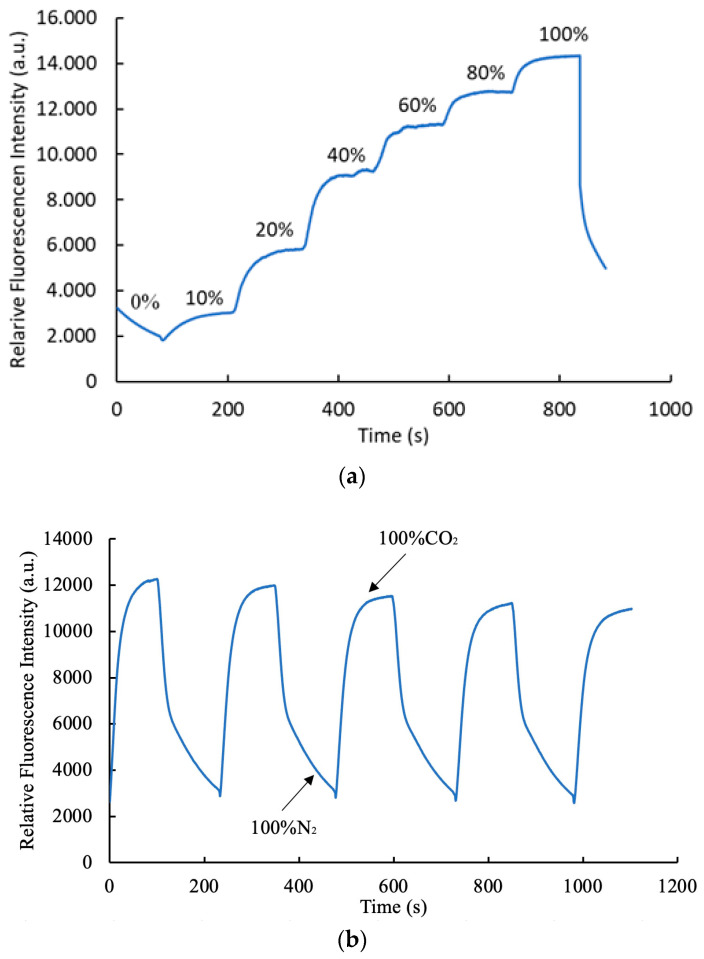
(**a**) Sensor response and (**b**) dynamic behavior of the carbon dioxide sensor.

The analysis of Figure 10a provides critical insights into the operational features of the carbon dioxide sensor based on an AAO substrate. The sensor exhibits a highly consistent and reproducible response to varying CO_2_ concentrations, as evidenced by the cyclic fluctuations in luminescence intensity. Over four consecutive cycles, the optical CO_2_ sensor achieved response and recovery times of 55 s and 120 s, respectively, during transitions between 100% CO_2_ and 100% N_2_ atmospheres. Figure 10b illustrates the dynamic performance of the carbon dioxide sensor under varying gas conditions. The sensor’s behavior was systematically analyzed by gradually increasing the CO_2_ concentration from 0% to 100%, transitioning the environment from pure nitrogen to pure carbon dioxide. During this process, the CO_2_ concentration increased over 120 s, facilitating the restoration of luminescence intensity in a nitrogen-enriched atmosphere. This response and recovery process is carried out in stages by dividing the concentration of carbon dioxide gas from 0% to 100%, with carbon dioxide gas applied for 2 min per gas concentration until the intensity of the glow reaches a stable saturation point. These results unequivocally demonstrate the optical sensor’s rapid responsiveness and efficiency, highlighting its suitability for real-time carbon dioxide concentration monitoring and detection applications.

### 3.9. The Analytical Selectivity Response of Optical Carbon Dioxide Sensor

**Figure 11:**
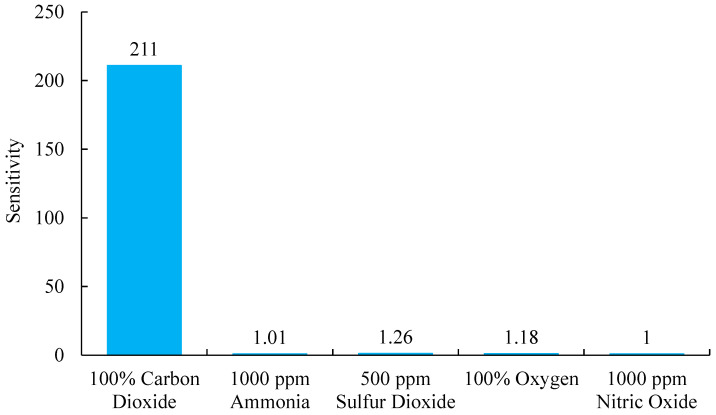
Selectivity of the optical carbon dioxide sensor in responding to different gases.

To guarantee accurate and reliable performance of the gas sensor across diverse gas mixture environments, rigorous selectivity testing is conducted to evaluate its capacity to differentiate among various gases. Selectivity tests using NH_3_, O_2_, SO_2_, and NO as target gases revealed the sensor’s capacity to accurately distinguish between these molecules. Figure 11 depicts the gas selectivity process, emphasizing the sensor’s capability to detect carbon dioxide. The concentration of CO_2_ in gas mixtures varies widely depending on the specific application, commonly measured in either percentage. As demonstrated, the sensor attained its peak sensitivity (*I*_100*%CO*2_/*I*_0*%CO*2_ = 211) concerning carbon dioxide at a 100% concentration, highlighting its exceptional capability in detecting and quantifying CO_2_, particularly in environments where it is the dominant gas. These results underscore the pronounced high selectivity of CdSe/ZnS QDs molecules embedded in the AAO substrate towards CO_2_. In contrast, the sensor exhibited significantly lower sensitivity to other gases, with ammonia and nitric oxide showing responses of only 1.01 and 1, respectively, at 1000 ppm. This reduced responsiveness may be attributed to differences in molecular structure or their interactions with the sensor’s material. Such observations are pivotal in assessing the sensor’s applicability and its performance limitations across diverse environmental monitoring contexts.

### 3.10. The Photostability of Optical Carbon Dioxide Sensor

**Figure 12:**
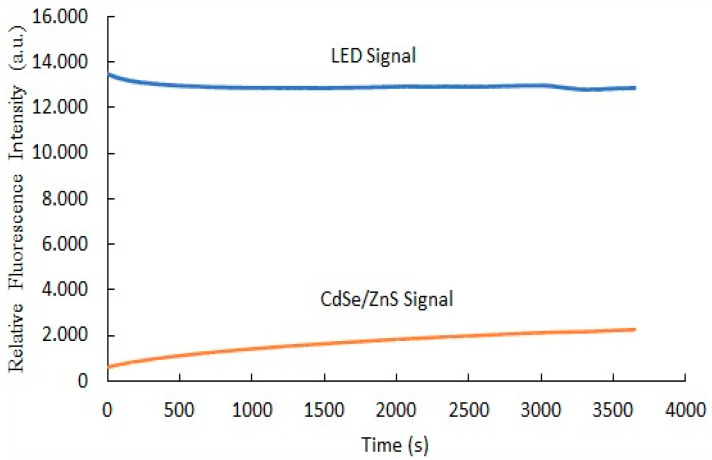
Photostability of optical carbon dioxide gas sensor.

Photostability testing is crucial for ensuring the long-term reliability of optical sensors under diverse real-world lighting conditions while maintaining their sensitivity and accuracy. Figure 12 illustrates that the blue curve, representing the LED signal, remained consistently stable throughout the 3600-s measurement period, indicating that the LED’s light output was steady during the test. In contrast, the orange curve, corresponding to the CdSe/ZnS quantum dots’ signal, displayed a gradual increase in fluorescence intensity over time, suggesting enhanced emission under continuous LED illumination. When excited by 405 nm wavelength blue light, the CdSe/ZnS QDs absorb the excitation light and emit fluorescence. This increase in fluorescence intensity implies that continuous LED exposure enhances the photoluminescence of the QDs. Photostability testing is essential in such cases, as prolonged light exposure may degrade fluorescent materials. By monitoring the signal over time, we can evaluate the photostability of the CdSe/ZnS quantum dots and their ability to endure sustained illumination. Overall, the findings demonstrate that despite the photoactivation effect, the sensor maintains a stable response to LED illumination, emphasizing its potential for long-term operation in carbon dioxide sensing applications.

## 4. Conclusions

This study successfully developed an optical carbon dioxide sensor employing a colorimetric approach, integrating Phenol Red and CdSe/ZnS quantum dots within a polyimide-butyl methacrylate matrix on an anodic aluminum oxide (AAO) substrate. The sensor exhibited a proportional increase in fluorescence intensity with rising CO_2_ concentrations. Stern–Volmer plot analysis revealed two distinct linear regions: in the 0–40% concentration range, fluorescence quenching displayed a strong linear correlation with an R^2^ value of 0.9706, while in the 40–100% range, the quenching followed a separate linear trend with an even higher R^2^ value of 0.9932, indicating superior sensitivity and reliability across both ranges. The sensor effectively detected CO_2_ with a broad linear range of 0–100% and demonstrated a sensitivity of 211. The observed shift of 0.1657 nm/% indicates a strong interaction between CO_2_ molecules, Phenol Red, and quantum dots within the AAO substrate. Its performance was characterized by a red peak emission at 570 nm when excited by a 405 nm LED, with response and recovery times of 55 and 120 s, respectively. Selectivity tests using NH_3_, O_2_, SO_2_, and NO as target gases confirmed the sensor’s exceptional selectivity, as it responded exclusively to CO_2_. Furthermore, the sensor exhibited good stability during continuous 1-h illumination, maintaining consistent performance. These results underscore the sensor’s robustness against variations in excitation light intensity, highlighting its suitability for CO_2_ detection in both medical and industrial applications.

## Figures and Tables

**Table 1:** A comparative analysis of material performance in optical CO_2_ sensing technologies.

pH-Sensitive Dye	Internal Reference Dye	Sensor Layer	Sensitivity	Sensing Signal	Ref.
Cresol Red in ethyl cellulose	Eu(tta)_3_ in polystyrene	Double layers	7.1	Intensity	[8]
Phenol Red in ethyl cellulose	Eu(tta)_3_ in polystyrene	Double layers	9.9	Intensity	[8]
Thymol blue in ethyl cellulose	Eu(tta)_3_ in polystyrene	Double layers	15.6	Intensity	[8]
α- naphtholphthalein in polyIBM	Tetraphenylporphyrin in polystyrene	Double layers	192	Intensity	[9]
α- naphtholphthalein in poly(TMSP)	Tetraphenylporphyrin in polystyrene	Double Layers	10.3	Intensity	[10]
α- naphtholphthalein in ethyl cellulose	Tetraphenylporphyrin in polystyrene	Double Layers	53.9	Intensity	[12]
Phenol Red in polyIBM	CdSe/ZnS QDs in polyIBM	Single layer	211	Wavelength shift/Intensity	This work

## Data Availability

The original contributions presented in this study are included in the article. Further inquiries can be directed to the corresponding author.
